# Entropy, Information, and Symmetry: Ordered is Symmetrical

**DOI:** 10.3390/e22010011

**Published:** 2019-12-19

**Authors:** Edward Bormashenko

**Affiliations:** Department of Chemical Engineering, Ariel University, Ariel 407000, Israel; edward@ariel.ac.il; Tel.: +972-074-729-68-63

**Keywords:** entropy, symmetry, ordering, Landauer principle

## Abstract

Entropy is usually understood as the quantitative measure of “chaos” or “disorder”. However, the notions of “chaos” and “disorder” are definitely obscure. This leads to numerous misinterpretations of entropy. We propose to see the disorder as an absence of symmetry and to identify “ordering” with symmetrizing of a physical system; in other words, introducing the elements of symmetry into an initially disordered physical system. We demonstrate with the binary system of elementary magnets that introducing elements of symmetry necessarily diminishes its entropy. This is true for one-dimensional (1D) and two-dimensional (2D) systems of elementary magnets. Imposing symmetry does not influence the Landauer principle valid for the addressed systems. Imposing the symmetry restrictions onto the system built of particles contained within the chamber divided by the permeable partition also diminishes its entropy.

## 1. Introduction

The notion of entropy, introduced by Rudolf Clausius [1,2], is considered to be one of the most important, but also the most abstract and least visualizable, quantities of physics [3,4]. Clausius (1867) coined the term entropy from the Greek word τροπη for transformation and change [5]. The classical thermodynamics macroscopic definition of the entropy change within a reversible process is $\Delta S=\frac{\Delta Q_{rev}}{T}$, where $\Delta Q_{rev}$ is the heat flow which takes place under the reversible process, and *T* is the temperature [5]. The statistical definition of the entropy is:

(1) $$S=k_{B}\ln\Omega ,$$

where Ω is the multiplicity of a certain state (i.e., the number of different configurations that a system defined by macroscopic variables could assume, or in other words, the number of states accessible to the system) [6,7,8,9]. The widespread illustrative interpretation of entropy is “the measure of disorder” in macroscopic systems built from a large number of particles [9]. However equating entropy with disorder was criticized recently [4,10,11]. Indeed, the multiplicity has a well-defined statistical meaning; whereas, “disorder” is an obscure notion, deeply rooted in human psychology, namely in the human perception of ordered and disordered patterns [12,13,14]. We propose the hypotheses that “ordering” may be strictly related to, and quantified by, symmetry. In turn, “chaos” or disorder is an absence of symmetry. It seems that the idea of symmetry as an adequate characteristic of ordering for various patterns was suggested by Yodogawa [15] and elaborated further by Zabrodsky, Avnir, and Peleg [16] as well as Petitjean [17]. We demonstrate that introducing symmetry into physical systems necessarily “orders” them and decreases their entropy. Symmetry considerations play a key role in modern science [18,19,20], giving rise to the conservation laws in physics, and serves as a dominant factor in quantum theory [21], crystallography [22,23], and condensed-matter physics. We show that it is crucial for constituting thermodynamics and informational properties of physical systems.

## 2. Symmetry and Entropy of Binary Systems

**Figure 1:**
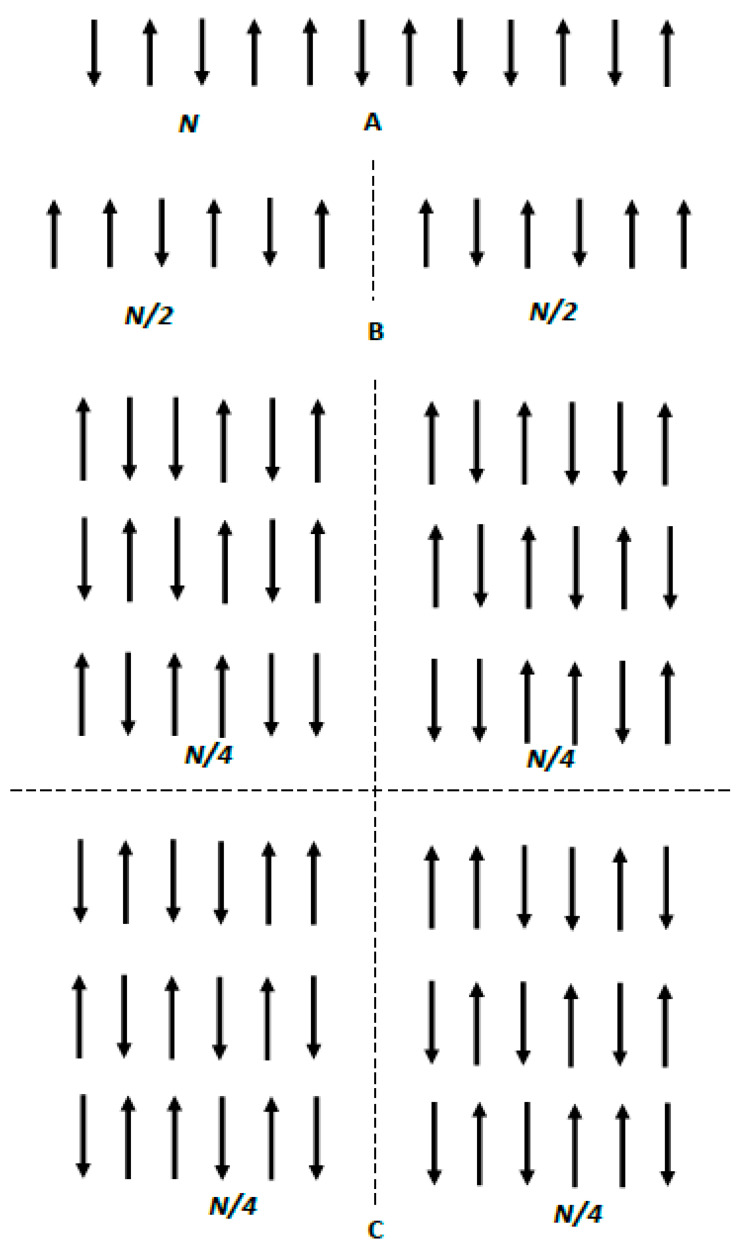
(**A**) The binary one-dimensional (1D) system of *N* non-interacting elementary magnets (the external magnetic field equals zero ($\vec{H}=0$). All of the up/down arrangements of the magnets are available. (**B**) The axis of symmetry shown with the dashed line restricts the number of available configurations of magnets. (**C**) Two-dimensional (2D) binary system of elementary magnets. Axes of symmetry shown with dashed lines restrict the available arrangements of the magnets.

First consider the binary one-dimensional (1D) system illustrated in Figure 1A. We assume that there are *N* separate and distinct sites fixed in a space and aligned as shown in Figure 1A [8]. Attached to each site is an elementary magnet, which can point only up or down. Thus, a binary, non-interacting system is created. The total number of arrangements of the *N* magnets is $\Omega =2^{N}$ if no external restrictions are imposed on the system and all the states are equally accessible to the system (only two possible states are available for the magnets). Thus, the entropy of the system is given by:

(2) $$S=k_{B}N\ln2$$

If the addressed binary system is in the thermal equilibrium with a thermal reservoir *T* and the external magnetic field equals zero ($\vec{H}=0$), then the modulus of the term of Helmholtz free energy $\left|F_{S}\right|$ arising from entropy contributions is supplied by:

(3) $$\left|F_{S}\right|=k_{B}TN\ln2$$

thus, illustrating the Landauer principle [24,25,26]. Indeed, if erasing one bit of information is performed under re-orientation of the single elementary magnet, the energy cost of such an erasure equals $k_{B}T\ln2$ in a strict accordance with the Landauer principle [24,25,26].

Now let us restrict the possible configurations of elementary magnets by introducing the symmetry axis, shown with the dashed line in Figure 1B. After introducing the symmetry axis, only symmetric configurations of the elementary magnets are available, as depicted in Figure 1B. The total number of arrangements of *N* magnets is $\Omega =2^{\frac{N}{2}}$. Hence, the entropy of the symmetrized, ordered, binary, non-interacting system is given by:

(4) $$S=k_{B}\frac{N}{2}\ln2$$

It is seen that introducing symmetry orders the binary system, and consequently decreases entropy. It seems plausible to equate “ordering” with symmetrizing of the system. If this identifying is accepted, the entropy is indeed the measure of disorder, in other words, an absence of symmetry. Obviously, the axis of symmetry may be replaced by the center of symmetry [18].

The modulus of the entropy-related term of the Helmholtz free energy denoted $\left|F_{S}\right|,$ for the symmetrized system shown in Figure 1B equals $\left|F_{S}\right|=k_{B}T\frac{N}{2}\ln2$. Thus, the Landauer principle remains untouched. Indeed, in this case erasing one bit of information is performed under simultaneous re-orientation of two symmetric elementary magnets, and the energy cost of such an erasure again equals $k_{B}T\ln2$ in accordance with the Landauer principle [24,25,26].

Consider now the two-dimensional (2D) system of elementary magnets depicted in Figure 1C. Generalization of the aforementioned approach for 2D systems is straightforward. If the system is not symmetrized (ordered) and the arrangement of elementary magnets is equally available, the entropy of the system is still given by $S=k_{B}N\ln2$. If the system is symmetrized and possesses two axes of symmetry, as shown in Figure 1C, its entropy is $S=k_{B}\frac{N}{4}\ln2$. Additional elements of symmetry in this 2D case (i.e., the axes or centers of symmetry) result in a larger decrease in the entropy of the system. This supports the idea that ordering (understood as symmetrizing) necessarily decreases the entropy.

**Figure 2:**
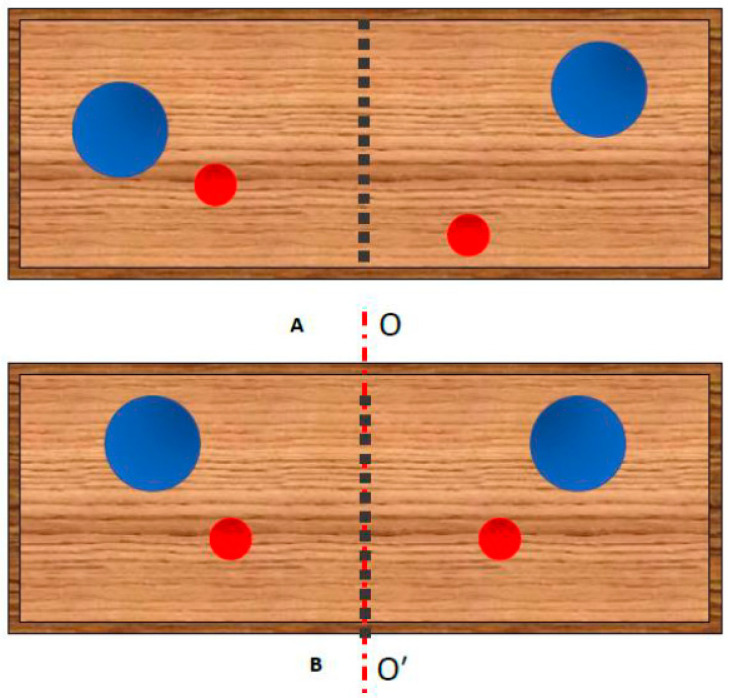
(**A**) Particles of two kinds (blue and red) are located within the chamber divided equally by the permeable partition. The arrangements at which two particles are simultaneously located at one side of the partition are permitted. (**B**) Particles of two kinds (blue and red) are located within the chamber divided equally by the permeable partition. Only the arrangements symmetric relatively to axis $OO^{\prime }$ shown with the red dashed line are permitted.

Consider one more example shown in Figure 2. Four molecules of two different kinds (i.e., blue and red ones) are located within a chamber divided equally by the permeable partition. The particles of the same kind are considered as indistinguishable. Consider the states, within which two molecules may be located simultaneously on one side of the partition. These states may be realized by three various arrangements (recall, that particles of the same sort are identical and indistinguishable). Hence, the entropy of such a system is $S=k_{B}\ln3$. Now let us introduce “ordering” into our system. Impose the demand that the particles should be necessarily located symmetrically relative to the axis $OO^{\prime }$ passing over the partition as depicted in Figure 2. Introducing symmetry diminishes the multiplicity to Ω=1. Consequently, the entropy of the symmetrized system of particles equals zero. The generalization of the suggested approach to the system of 2*N* particles is straightforward.

It is noteworthy, that the notion of the Voronoi entropy fails to quantify the ordering of points constituting the 2D pattern [27,28,29]. It was demonstrated that the symmetric patterns may be characterized by the values of the Voronoi entropy being very close to those inherent to random ones [30].

## 3. Conclusions

Misinterpretations of entropy are ubiquitous among scientists and non-scientists [4,11]. The widespread interpretation of entropy is “the measure of disorder” [4,11]. However, this understanding of entropy was criticized recently and equating entropy to “disorder” or “chaos” was considered a misconception [4,11]. Indeed, “disorder” and “chaos” are obscure notions, actually rooted in human psychology [12,13,14], that can hardly be accurately defined. We demonstrate how this misconception may be avoided. The misunderstanding is eliminated if “ordering” is identified with symmetrizing the system. Thus, “chaos” or “disorder” is understood as an absence of symmetry. We demonstrate that introducing elements of symmetry orders the system and consequently diminishes its entropy. The idea is illustrated through a binary system built from elementary magnets where 1D and 2D exemplifications of the binary systems are presented. We also demonstrate that introducing symmetry does not influence the Landauer principle for binary systems. We also considered the system built of particles contained within the chamber divided by the permeable partition. Again, imposing symmetry restrictions diminished the entropy of the system. To be rigorous, the author concludes that it is possible that other pathways of ordering physical systems, apart from imposing elements of symmetry, exist; these alternative pathways call for additional physical insights.
